# Liver Resection: Prolonged Inflow Occlusion in Human Cirrhotic Livers

**DOI:** 10.1155/1996/45405

**Published:** 1996

**Authors:** Bengt Jeppsson

**Affiliations:** Department of Surgery Lund University Hospital Lund S-221 85 Sweden

## Abstract

To evaluate the tolerance of the cirrhotic liver to extended warm ischaemia, 47 patients with cirrhosis who underwent liver,resection over a 4-year period were studied retrospectively. Three groups of patients were identified. In group 1 (14 patients) liver resection was performed under conditions of portal triad occlusion ranging from 50 to 75 (mean 57.1) min. Group 2 (12 patients) was treated with portal occlusion for a period ranging from 30 to 42 (mean 33.1) min. Group 3 comprised 21 patients who underwent  hepatectomy using conventional techniques. Mean blood loss was significantly reduced by portal triad occlusion (819 ml in group 1,523 ml in group 2) compared with the conventional techniques (1652 ml in group 3) (P<0.05, group 1 versus group 3; P<0.01, group 2 versus group 3). Hospital death occurred in three of the 21 patients in group 3 but in no patient who underwent portal triad occlusion. The morbidity rate was lower in the two occlusion groups (four of 26 patients) than in group 3 (six of 21). Bilirubin metabolism was substantially better after surgery in the occlusion groups (P<0.05, groups 1 and 2 versus group 3 at day 14). Although the serum levels of transaminases were significantly raised until day 3 in patients undergoing long term occlusion, the cirrhotic liver withstood the ischaemia-reperfusion insult, as assessed by changes in hepatic microcirculation, lipid peroxidation and the morphology of hepatic sinusoids. It is concluded that prolonged ischaemia during liver resection can be sustained in patients with cirrhosis and without high-risk factors.


separating the right and left liver and dividing the liver along the right margin of Segment IV so as to approach the caudate intra-parenchymally from above. A combination of this technique with the methods describe above may be useful in such large lesions[4]. It is perhaps a minor criticism of this contribution that the authors do not mention this later contribution from Japan.

We have now resected a total of 21 complete caudate resections, 4 of whom underwent isolated caudate lobectomy[5]. The most common diagnosis was metastatic colorectal cancer in nine patients and the most common procedure was an extended left hepatic lobectomy with en bloc caudate lobectomy. The median operative time was five hours and the median blood loss was1,160 ml. Only one patient required intensive care and went on to die of liver failure. In none of our cases was vascular isolation employed but an intermittent Pringle maneuver combined with temporary occlusion of the left and middle hepatic veins was the method of choice[5].

In short, although only two cases of isolated hepatic lobectomy are reported this paper is of importance since there are but few reports of isolated caudate lobe resection, the most recorded by an author being the three cases reported by Colonna *et al.*[6] and the four cases referred to and submitted for publication by our own group.

There is no doubt that the development of hepatic resectional techniques and the understanding of the anatomy of the caudate lobe has resulted in an ability to resect the isolated caudate lobe safely, and that the addition of caudate lobectomy to major liver resection does not add significantly to the morbidity or mortality of the procedure.

### REFERENCES

1. Kumon M. (1985) Anatomy of the caudate lobe with special reference to portal vein and bile duct (in Japanese). Acta Hepatol Jpn 26: 1193–9.
2. Couinaud C. Couinaud C.,ed. (1989) Surgery anatomy of the liver revisited.Paris:Maugein& Ci 123–34.
3. Lerut J, Gruwez JA, Blumgart LH.(1990) Resection of the caudate lobe of the liver. Surg Gynecol Obstet 171: 160–2.
4. Yamamoto J, Takayama T, Kosuge T, et al. (1992) An isolated caudate lobectomy by the transhepatic approach for hepatocellular carcinoma in cirrhotic liver. Surgery 111: 699–702.
5. Bartlett D, Fong Y, Blumart LH. (1995) Complete resection of the caudate lobe of the liver-technique and results. *British Journal of Surgery,* In Press.
6. Colonna JO, Shaked A, Gelabert HA, Busuttil RW. (1993) Resection of the caudate lobe through "bloody gultch". Surg Gynecol Obstet 176: 401–2.

Leslie H Blumgart, MD, FACS, FRCS
Chief:Hepatobiliary Service
Memorial Sloan-Kettering Cancer Center
1275 York Avenue
New York, New York 10021
United States of America

### LIVER RESECTION: PROLONGED INFLOW OCCLUSION IN HUMAN CIRRHOTIC LIVERS

### ABSTRACT

*Kim, Y.I., Nakashima, K., Tada, I., Kawano, K. and Kobayashi, M. (1993) Prolonged Ormothermic Ischaemia of Human Cirrhotic Liver during Hepatectomy: A Preliminary Report. Br J Surg, 80: 1566–1570.*

**To evaluate the tolerance of the cirrhotic liver to extended warm ischaemia, 47 patients with cirrhosis who underwent liver resection over a 4-year period were studied retrospectively. Three groups of patients were identified. In group 1 (14 patients) liver resection was performed under conditions of portal triad occlusion ranging from 50 to 75 (mean 57.1) min. Group 2 (12 patients) was treated with portal occlusion for a period ranging from 30 to 42 (mean 33.1) min. Group 3 comprised 21 patients who underwent hepatectomy using conventional techniques. Mean blood loss was significantly**

**reduced by portal triad occlusion (819 ml in group 1, 523 ml in group 2) compared with the conventional techniques (1652 ml in group 3 ) ($P<0.05$, group 1 versus group 3; $P<0.01$, group 2 versus group 3). Hospital death occurred in three of the 21 patients in group 3 but in no patient who underwent portal triad occlusion. The morbidity rate was lower in the two occlusion groups (four of 26 patients) than in group 3 (six of 21). Bilirubin metabolism was substantially better after surgery in the occlusion groups ($P<0.05$, groups 1 and 2 versus group 3 at day 14). Although the serum levels of transaminases were significantly raised until day 3 in patients undergoing long term occlusion, the cirrhotic liver withstood the ischaemia-reperfusion insult, as assessed by changes in hepatic microcirculation, lipid peroxidation and the morphology of hepatic sinusoids. It is concluded that prolonged ischaemia during liver resection can be sustained in patients with cirrhosis and without high-risk factors.**

KEY WORDS: Liver inflow ischaemia liver resection liver cirrhosis pringle manoeuvre

### PAPER DISCUSSION

Liver resection is risky for patients with chronic liver disease because the diseased liver has a decreased regenerative capacity and an increased incidence of intraoperative and postoperative haemorrage due to coagulopathy and portal hypertension[1]. The amount of intraoperative haemorrage is correlated with postoperative morbidity and mortality[2], therefore all measures should be taken to minimise blood loss during surgery. To keep bleeding to a minimum, intrahepatic blood flow has to be arrested. The question then arises how tolerant the cirrhotic liver is to hypoxia. Since hepatic failure in cirrhotic patients often occurs after a massive bleeding from e.g.varices, this empiric fact suggests that the cirrhotic liver is more vulnerable to hypoxia than the normal liver. The regenerating nodules in the cirrhotic liver are more dependant on arterial blood supply than portal supply and this may render them more vulnerable to hypoxia. Few studies have been performed evaluating ischaemic insults in cirrhotic livers.

Several clinical studies have convincingly shown that the cirrhotic liver can tolerate ischemia quite well [3,4]. This elegant study by Kim *et al.* addresses this problem. They have studied 26 patients with liver cirrhosis undergoing liver resection under portal triad occlusion and compared the results to 21 patients with cirrhosis undergoing liver resection with standard technique without occlusion. The patients were divided in two groups, one with a median occlusion time of 57.1 minutes and another with 33.1 minutes. There was no mortality in patients undergoing portal triad occlusion. The morbidity rate was also lower in the two occlusion groups (4/26) compared to the group with standard resection (6/21). Serum levels of transminases were significantly raised in patients with long term occlusion but apart from that, the cirrhotic liver seems to withstand the ischemia/reperfusion insult well. This was studied by tissue blood flow and malondialdehyde levels during ischemia/reperfusion. These two measurements did not indicate any severe ischaemic damage. One explanation for the good tolerance of the liver to ischemia is the retrograde flow of blood from the vena cava in to the hepatic veins alternating with pressure differences in the thoracic cavity. This backflow may contribute to the maintenance of liver function.

There are a few points that I would like to discuss in relation to this paper. Firstly, the benefit of reducing blood loss. In this study there was a significant reduction in blood loss in patients undergoing occlusion compared to those operated with a conventional technique (819 ml vs 1652 ml). Similar findings have been found by others[3] and the postoperative morbidity was, in the latter study, also related to the amount of blood loss. So therefore all measures that can be taken in order to reduce blood loss are of benefit for the postoperative course. Besides inflow occlusion, in situ or surface liver cooling may also further reduce the bleeding as has been shown in another paper[5].

The second issue is the tolerance of the cirrhotic liver to ischemic damage. One most state that there is quite substantional clinical evidence that the cirrhotic liver may tolerate up to more than an hour of warm ischemia. There is also experimental evidence that although the injury is somewhat more pronounced in cirrhotic livers the impact of this in relation to regeneration after resection in minot[6,7]. In the experimental study on regeneration[7] it is found that the detrimental effect on hepatic regeneration was more due to portal pooling and reperfusion of portal blood than ischemia/reperfusion by itself.

One problem in the preoperative evaluation of patients with liver cirrhosis is how to estimate the amount of ischaemia that can be inflicted on the liver with no harm. The patient group with liver cirrhosis comprises a wide range of functional reserve in relation to operative tolerance to ischemia. This is particularly difficult since we do not know which cell types in the liver are more prone to hypoxia. One group of patients which seems to be at particularly high risk for morbidity is patients with cholestasis. In this group of patients with cirrhosis and cholestasis preoperative drainage prior to resection may be indicated.

In conclusion, patients with cirrhosis can undergo hepatic resection with inflow occlusion with reduced blood loss and thereby reduced morbidity without inflicting any significant ischaemic damage to the liver, if ischaemia time is of one hour duration or less.

### REFERENCES

1. Nishimura T, Nakahara M, Kobayashi S, Hotta I, Yamawaki S, Marui Y: (1992) Ischemic injury in cirrhotic livers: An experimental study of the temporary arrest of hepatic circulation. *Journal of Surgical Research* **53**: 227–233.
2. Nagorney DM, van Heerden JA, Ilstrup DM, Adson MA: (1989) Primary hepatic malignancy: Surgical management and determinants of survival. *Surgery* **106**: 740–749.
3. Nagasue N, Uchida M, Kubota H, Hayashi T, Kohno H, Nakamura T: (1995) Cirrhotic liver can tolerate 30 minutes ischaemia at normal environmental temperature. *European Journal of Surgery* **161**: 181–186.
4. Emond J, Wachs M, Renz J, Kelley S, Harris H, Roberts J, Ascher N, Lim R: (1995) Total vascular exclusion for major hepatectomy in patients with an abnormal liver parenchyma. *Archives of Surgery* **130**: 824–831.
5. Kim IY, Kobayashi M, Nakashima K, Aramahi M, Yoshida T, Mitarai Y: (1994) In situ and surface liver cooling with prolonged inflow occlusion during hepatectomy in patients with chronic liver disease. *Archives of Surgery* **129**: 169–624.
6. Usami M, Furuchi K, Shiroiwa H, Saitoh Y: (1994) Effect of repeated portal-triad cross-clamping during partial hepatectomy on hepatic regeneration in normal and cirrhotic rats. *Journal of Surgical Research* **57**: 541–548.

Bengt Jeppsson
Department of Surgery
Lund University Hospital
S-221 85 LUND
Sweden

### IMPROVED RESULTS FOR RESECTION OF PERIAMPULLARY ADENOCARCINOMA

### ABSTRACT

*Andersen, H.B., Baden, H., Brahe, N.E.B. and Burcharth, F. (1994) Pancreaticoduodenectomy for periampullary adenocarcinoma Journal of the American College of Surgeons 179: 545–552.*

***Background:*** **This study evaluates the indications for and effects of pancreaticoduodenectomy (102 patients) or total pancreatectomy (15 patients) with extensive lymph node dissection performed upon 117 patients for treatment of periampullary adenocarcinoma.**
***Study Design:*** **Presenting symptoms and postoperative morbidity and mortality rates were recorded. Cumulative survival rates were evaluated in relation to origin, size, and staging of tumor. Postoperative follow-up of clinical symptons was done after one year.**
***Results:*** **The postoperative mortality rate after Whipple's operation was 8 percent (eight patients). The median survival period was 1.1 year and the overall five year survival rate**

